# Municipal mortality due to thyroid cancer in Spain

**DOI:** 10.1186/1471-2458-6-302

**Published:** 2006-12-15

**Authors:** Virginia Lope, Marina Pollán, Beatriz Pérez-Gómez, Nuria Aragonés, Rebeca Ramis, Diana Gómez-Barroso, Gonzalo López-Abente

**Affiliations:** 1 Cancer and Environmental Epidemiology Area, National Center for Epidemiology, Carlos III Institute of Health, Madrid, Spain

## Abstract

**Background:**

Thyroid cancer is a tumor with a low but growing incidence in Spain. This study sought to depict its spatial municipal mortality pattern, using the classic model proposed by Besag, York and Mollié.

**Methods:**

It was possible to compile and ascertain the posterior distribution of relative risk on the basis of a single Bayesian spatial model covering all of Spain's 8077 municipal areas. Maps were plotted depicting standardized mortality ratios, smoothed relative risk (RR) estimates, and the posterior probability that RR > 1.

**Results:**

From 1989 to 1998 a total of 2,538 thyroid cancer deaths were registered in 1,041 municipalities. The highest relative risks were mostly situated in the Canary Islands, the province of Lugo, the east of La Coruña (Corunna) and western areas of Asturias and Orense.

**Conclusion:**

The observed mortality pattern coincides with areas in Spain where goiter has been declared endemic. The higher frequency in these same areas of undifferentiated, more aggressive carcinomas could be reflected in the mortality figures. Other unknown genetic or environmental factors could also play a role in the etiology of this tumor.

## Background

At the European level, Spain ranks midway in terms of thyroid cancer (TC) incidence. This is a tumor that is far more frequent among women than among men. In recent years, incidence rate per 100,000 population has increased from 1.73 to 2.22 in the periods 1993–1996 [1] and 1997–2000 [2] in men, and from 3.63 [1] to 5.69 [2] per 100,000 in women. In the 1990s, mortality due to this tumor in males registered a statistically significant [2] mean annual increase of 1.21%, which in 2004 rose to a rate, standardized to the European population, of 0.33 per 100,000 population [3]. In women, however, TC mortality declined significantly by an average of 0.39% per annum [2], until reaching a rate of 0.49 per 100,000 population in 2004 [3]. Prevalence attributable to cases diagnosed in the preceding 5 years has reached a figure of 1559 cases in men and 4901 in women [4]. In Spain, TC is the tumor with the highest survival rate in women (86% at 5 years), and ranks second after testicular cancer in men (82%) [5].

Most thyroid tumors are epithelial and are classified into well-differentiated thyroid tumors (which include, in order of frequency, papillary, follicular, and oncocytic carcinoma subtypes), medullary tumors (with a genetic component) and anaplastic carcinomas (the most aggressive type) [6]. The best-known TC risk factor is exposure to ionizing radiation, whether through therapeutic irradiation or environmental pollution. Nevertheless, there are also epidemiologic studies that associate TC with clinical history of benign thyroid diseases, such as goiter or adenomas [7,8], hormonal and reproductive factors [8-12], dietary factors (such as iodine deficiency or high consumption of goitrogenic foods) [8,13-16], and genetic factors [17].

Thyroid cancer mortality in Spain is not distributed uniformly, being more pronounced in iodine-deficient geographic areas, mainly in the north-west of the country and the Pyrenees [18]. Spatial epidemiology is a relatively new discipline that seeks to describe, quantify and explain geographic variations in diseases and their relationship with factors generally of environmental origin. Advances in computation techniques, the ever-greater availability of georeferenced incidence and mortality data at different levels, the development of new statistical and epidemiologic methods for cluster research [19,20], and the growing public interest in the effects of environmental pollution, are reinforcing the potential of this discipline.

Analysis of small areas (generally districts or municipalities) improves the interpretation of results and the capacity to detect local effects linked to environmental problems, while reducing ecologic biases [21]. This study sought to study municipal distribution of TC mortality in Spain using these techniques, and to discuss the possible relationship between such distribution and the risk factors outlined above.

## Methods

Individual entries for the period 1989–1998, corresponding to deaths in towns and cities throughout Spain due to thyroid cancer (International Classification of Diseases, ICD-9 rubric 193), were used as the case source. These data were supplied by the National Statistics Institute (NSI) (*Instituto Nacional de Estadística*) for the production of a municipal cancer mortality atlas.

The municipal populations, broken down by age group (18 groups) and sex, were drawn from the 1991 census and 1996 municipal voters rolls. These years correspond to the midway points of the two quinquennia that comprise the study period (1989–1993 and 1994–1998). The person-years for each five-year period were estimated by multiplying these populations by 5.

Standardized mortality ratios (SMRs) were calculated as the ratio between observed and expected deaths. For the calculation of expected cases, the overall Spanish mortality rates for the above two 5-year periods were applied to each town's person-years by age group, sex and quinquennium.

To draw up these maps, smoothed municipal relative risks (RRs) were calculated using the autoregressive conditional model proposed by Besag, York and Mollié. This model was introduced by Clayton and Kaldor (1987), developed by Besag, York and Mollié [22], and subsequently applied in the field of ecologic studies [23]. Such models are based on fitting spatial Poisson models with two random-effect terms that take the following into account: a) the effects which vary in a structured manner in space (municipal contiguity); and, b) a component that models the effects which vary among municipalities in an unstructured manner (municipal heterogeneity) [24]. The model takes the following form

*O_i _*~ *Po*(*E_i _*λ*_i_*)

log(λ*_i_*) = α + *h_i _*+ *b_i_*

where: λ_i _is the relative risk in area I; O_i _is the number of deaths in area I; *α *is the intercept; E_i _are the expected number of cases; h_i _is the municipal heterogeneity term; and b_i _is the spatial term.

The models were fitted using Bayesian Markov Chain Monte Carlo simulation methods with improper priors [25]. Posterior distributions of relative risk were obtained using WinBugs [26]. The criterion of contiguity needed for the model was adjacency of municipal boundaries. Convergence of the simulations was verified using the BOA (Bayesian Output Analysis) R program library [27]. Given the great number of parameters of the models, the convergence analysis was performed on a randomly selected sample of 10 towns and cities, taking 4 strata defined by municipal size. Convergence of the estimators was achieved before 100,000 iterations. In the present study, a "burn-in" (iterations discarded to ensure convergence) of 300,000 iterations was performed, and the posterior distribution was derived using 5,000 iterations. The CPU time on a Pentium 2 GHz was 18 hours.

A Geographic Information System (GIS) was used to create municipal maps of SMRs, smoothed RR estimates, and the posterior probability that RR > 1. In the case of this last-mentioned indicator, we applied Richardson's criterion [21], which recommends that probabilities over 0.8 be deemed significant.

To facilitate the location of regions cited in this paper, a map showing the respective Spanish Autonomous Regions and provinces is attached (see Additional data file 1).

## Results

From 1989 through 1998, a total of 2538 TC deaths were registered in Spain (809 in men and 1729 in women). Cases were registered in 1041 towns and cities. Using these data we were able to compile and ascertain the posterior distribution of relative risk on the basis of a single spatial model that included all of Spain's 8077 towns and cities and the 46398 adjacencies existing between them. The maps in the Annex show 8225 rather than the 8077 NSI-coded towns and cities, due to the fact that there are 148 stretches of territory in Spain which are regarded either as state-owned (communally exploited areas traditionally known as *parzonerías*, and military zones), municipal common land (pastures, common and heath), or land belonging jointly to two or more municipalities (*mancomunidad*; union or league of towns). To allow the coding of terrain not identified by the NSI, the code of the nearest town was allocated to such areas so that the spatial analyses could be performed.

**Figure 1:**
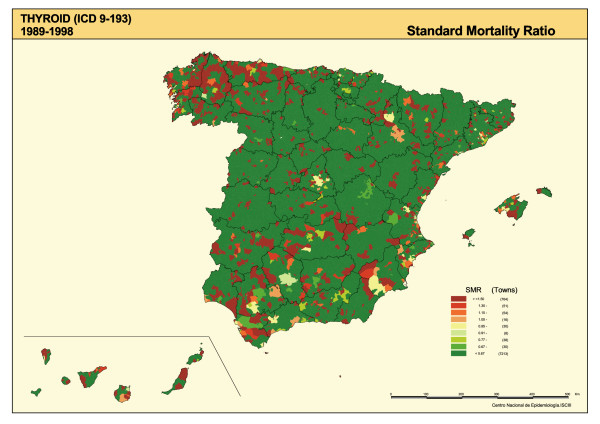
Municipal distribution of thyroid cancer mortality. Standardized mortality ratios (SMRs). Spain 1989–1998.

**Figure 2:**
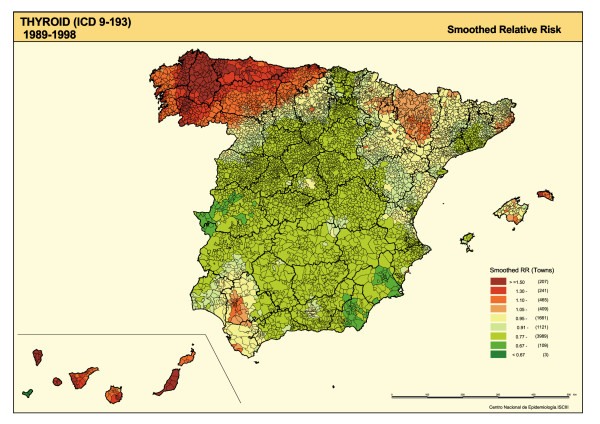
Municipal distribution of thyroid cancer mortality in Spain. Distribution pattern of smoothed relative risk (RR) under the BYM model. Spain 1989–1998.

**Figure 3:**
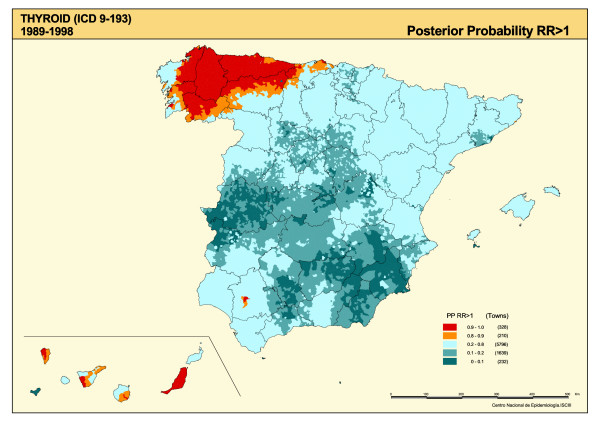
Municipal distribution of thyroid cancer mortality. Posterior probability of RR being greater than 1. Spain 1989–1998.

The SMRs are shown in Figure 1, the distribution of smoothed RRs for TC is plotted in Figure 2, and the posterior probability of RR being greater than 1 is depicted in Figure 3. The SMR map shows the polarization of the distribution towards its extremes (towns with and without cases), with no specific pattern being clearly discernable. The "noise" present in this map, deriving from the instability of the indicator, was eliminated by the smoothing procedure (Figure 2).

Notable in the smoothed map (Figure 2) is the concentration of higher TC mortality in the north-west of the country, mainly in the province of Lugo, the east of La Coruña (Corunna), and western areas of Asturias and Orense. With the exception of the Canary Islands, there was no other region with such high mortality values.

Table 1 reports information on the most representative towns and cities with excess TC mortality. The selection criteria chosen were as follows: any municipality with at least 3 observed cases, a difference between observed and expected cases of 2 or more, an RR of over 1.2, and a posterior probability of 0.8 or more of RR exceeding 1. A total of 39 towns and cities, situated in 12 provinces, met these criteria. In all, 75% of highlighted localities lay in the north-west of the country and were mainly concentrated in 2 Autonomous Regions, namely, Galicia and Asturias. Outside this area, attention should be drawn to the raised risk observed in some towns in the Canary Islands and in the City of Seville, with 62 observed versus 40 expected cases. Puerto del Rosario (in the Canaries), followed by El Ferrol (in Galicia), were the towns with the highest RRs in Spain.

**Table 1:** Selected towns and cities with excess thyroid cancer mortality. *Spain 1989–1998.

**Autonomous Region**	**Province**	**City or Town**	**Obs**	**Exp**	**SMR**	**Smoothed RR**	**P (RR > 1)**
Andalusia	Seville	Seville	62	39.65	1.56	1.298	0.984
Asturias	Asturias	Corvera de Asturias	3	0.91	3.30	1.489	0.978
		Gijón	27	18.89	1.43	1.415	0.991
		Langreo	7	3.96	1.77	1.501	0.979
		Lena	6	1.08	5.57	1.582	0.994
		Mieres del Camino	6	3.97	1.51	1.481	0.995
		Oviedo	24	14.03	1.71	1.522	0.998
		Tineo	4	1.34	2.99	1.562	0.991
Canary Islands	Palmas	Puerto del Rosario	3	0.51	5.91	3.955	0.962
		Santa Lucía	5	1.10	4.54	1.626	0.925
		Telde	6	2.91	2.06	1.337	0.863
	Santa Cruz de Tenerife	Arona	4	0.82	4.87	1.692	0.935
		Guia de Isora	4	0.54	7.45	1.621	0.940
		Paso (El)	4	0.46	8.64	1.734	0.920
		Santa Cruz de Tenerife	14	10.64	1.32	1.289	0.856
Cantabria	Santander	Camaleño	4	0.14	28.99	1.584	0.982
Castile & Leon	Leon	Cistierna	3	0.40	7.43	1.338	0.929
Catalonia	Barcelona	Calella	5	0.86	5.85	1.410	0.839
	Gerona	Sant Feliu de Guixols	4	1.18	3.40	1.452	0.836
Galicia	Corunna	Corunna	21	15.95	1.32	1.288	0.918
		Ferrol	13	6.17	2.11	1.963	0.995
		Melide	3	0.72	4.18	1.770	0.998
		Mesia	4	0.32	12.66	1.644	0.995
		Naron	5	1.96	2.55	1.768	0.990
		Oza dos Rios	3	0.34	8.72	1.691	0.994
		Porto do Son	3	0.78	3.85	1.327	0.850
	Lugo	Barreiros	3	0.41	7.35	1.804	0.992
		Begonte	3	0.46	6.59	1.798	0.999
		Cervantes	3	0.29	10.42	1.607	0.996
		Folgoso do Courel	3	0.24	12.61	1.595	0.999
		Friol	5	0.55	9.17	1.824	0.999
		Mondoñedo	3	0.65	4.61	1.747	0.997
		Monforte de Lemos	5	2.03	2.47	1.522	0.987
	Orense	Carballiño (O)	4	1.03	3.87	1.620	0.994
		Ourense	11	7.01	1.57	1.556	0.996
		Ribadavia	4	0.53	7.50	1.550	0.985
	Pontevedra	Silleda	3	0.94	3.21	1.460	0.968
		Vigo	24	16.10	1.49	1.308	0.934
		Vilagarcia de Arousa	4	1.96	2.04	1.385	0.910

Obs = observed deaths; Exp = expected deaths; SMR = standard mortality ratio; Smoothed RR = smoothed relative risk; P (RR > 1) = posterior probability that RR > 1.

* Towns and cities that fulfill the following criteria: (Obs – Exp) > = 2; Obs >= 3; RR > 1.2 y (P(RR) > 1) >= 0.8.

## Discussion

The results of this study reveal a higher risk of death due to TC in the north-west of the country and the Canary Islands. The pattern observed in this study is in line with an earlier study undertaken at a provincial level, in which the provinces of Lugo, Asturias, Las Palmas de Gran Canaria, and Corunna featured among the 5 with highest mortality in the period 1975–1995 [18]. Analysis at a municipal level has however led to the detection of small areas of risk, which had been masked at a provincial level. When separate analyses were performed for men and women, a similar geographic pattern was in evidence for both sexes (data not shown).

Standard errors of SMR are inversely dependent on the number of expected cases. This implies that the most extreme SMRs occur in small population areas and are based on a small number of cases. Maps of unsmoothed SMRs are therefore dominated by "green areas" (low RRs) and "red areas" (high RRs), most likely reflecting random variation. The smoothed map, however, tends to eliminate part of the random variability, solely highlighting those areas in which the risk is concentrated, so that a town with no TC deaths could be regarded as having excess risk due to the elevated risk of its neighbors and vice-versa. As a result, this could give rise to false positives (enhancing the risk of some towns), as occurs in certain Galician areas, or false negatives (attenuating the risk of others), as occurs in certain areas of Badajoz, Ciudad Real, Albacete, Cordoba, Jaen and Murcia.

When it comes to interpreting the results, a number of limitations must be borne in mind. In the first place, mortality is not the best indicator of the frequency of appearance of TC, since this tumor registers a high rate of survival. Yet, the lack of a cancer registry for the country as a whole, forces us to use this data source. Moreover, TC includes different histological types with different degrees of aggressiveness, hence mortality possibly reflects the incidence of the most aggressive tumor types in particular. Given that the highest risk appear in remote rural areas, mortality could also reflect differential accessibility to health care. Finally, TC, like all solid tumors, has a long latency period, so that the mortality pattern observed in our study would thus reflect the distribution of possible risk factors present in Spain some decades ago.

As stated above, the results of this study essentially reveal a greater risk of TC in the north-west of mainland Spain and in the Canary Islands, both traditionally areas of endemic goiter. The first descriptions of endemic goiter in Spain date back to the latter part of the 19th century. In 1927, Dr. Gregorio Marañón first reported the problem and, thanks to the strong pressure subsequently brought to bear by a certain scientific societies and groups of experts, a Royal Decree was passed in 1983 requiring the iodization of packaged table salt. In 1990, Spain committed itself to eradicating iodine deficiency by the year 2000. Yet, there is still no nationwide legislation governing the universal iodization of salt and Spain suffers from mild-moderate iodine deficiency [28], with the more severely affected areas being located in Galicia and certain parts of the Province of Leon [29].

Among the possible factors causing goiter are: first, a low intake of iodine (basically present in saltwater fish and seafood); second, congenital failures in the biosynthesis of thyroid hormones; and, third, antithyroid or goitrogenic substances, which interfere with the correct assimilation of iodine and are found in foods (thiocyanates, isothiocyanates and thioglucosides present in vegetables such as cabbage, Brussels sprouts and cauliflower) and drinking water (contaminated with *E. Coli *or compounds of a geologic origin) [30]. Some authors are, however, of the opinion that the role of goitrogenic substances in the etiology of endemic goiter is very limited [31].

Insofar as the relationship between TC and endemic goiter is concerned, iodine deficiency has been described as possibly having oncogenic power due to sustained hyperstimulation of the thyroid induced by elevated levels of thyroid stimulating hormone (TSH). This can stimulate clones of follicular cells with an altered phenotype that renders them prone to proliferate until autonomous growth clones appear which may, in turn, give rise to cancer [32]. Although there are no data to show that overall prevalence of TC is greater in areas with endemic goiter [33], some studies reported a higher risk among subjects who live in such areas [34-36]. Furthermore, several papers have described that iodine deficiency and iodine excess may induce changes in the distribution of histological types, inasmuch as areas with sufficient iodine have been associated with a higher prevalence of papillary carcinoma whereas areas with iodine deficiency are associated with a higher frequency of follicular and anaplastic thyroid cancer [33,37,38]. A possible explanation for this phenomenon might reside in the fact that in endemic goiter areas, i.e., traditionally mountainous areas having a lower level of socio-healthcare development, differentiated TC might not have been diagnosed in time. While there is no clear evidence that remedying iodine deficiency by iodine prophylaxis reduces TC incidence/mortality [37], some studies have nonetheless shown that iodine prophylaxis results in less aggressiveness in its biologic behavior and a regression in the frequency of advanced tumor stages within the same histologic type [33].

In our results, we have detected a higher risk of death due to TC in Galicia. Rural Galicia has historically been one of the endemic goiter regions in Spain. Up until relatively recently, it was not unusual to encounter subjects with large-sized goiters in endemic areas inland and along the main access routes into Galicia (via Orense or Lugo) [39]. Among the factors that possibly favored this endemic disease are: low iodine content in the water; low consumption of fish in mountainous areas; lack of road and rail transport and telecommunications; and a diet based on plants of the genus *Brassica *[40,41]. It is worth mentioning that habitants of Galician consume much more green leafy and other vegetables (mainly, different varieties of turnip top, Brussels sprouts and cabbage) than the national average [42]. Furthermore, low levels of selenium in the diet can alter the hormonal thyroid metabolism, either through formation of free radicals or through inhibition of the deiodinase that converts thyroxine (T4) into triiodothyronine (T3) [43,44]. Indeed, some studies addressing cancer in human beings suggest that sub-normal selenium levels in the diet could raise the risk of developing cancer [45]. As a consequence of its acid soils, Galicia is regarded as a selenium-poor area; levels in newborns are low and increase with age [46]. Of all the Galician provinces, Lugo registered the highest mortality rates. This was also the province where Garrido et al. reported the highest prevalence of goiter in Galicia [39], a complaint that continues to be frequent despite the institutional campaign that was launched in 1985, advocating the use of iodized salt [47,48].

Asturias is classified as a region having Grade I endemic goiter (i.e., palpable though not visible when the neck is normally at rest) [29]. In 1983, an iodized salt campaign targeted the entire Asturian population and made its use compulsory in all school canteens. After 18 years of iodine prophylaxis, prevalence of goiter has fallen appreciably [49]. In 1985, Martínez Rodríguez et al, in a study on TC in Asturias, detected a high proportion of undifferentiated forms. They observed that anaplastic forms were more frequent in endemic goiter areas, and in general, that all types were more common in cases where patients were or had been residing in such areas; but if the same patients were to be transferred to urban/industrial areas they would preferably develop a differentiated carcinoma [50]. In this respect, the higher TC mortality observed in the western region of Asturias could be explained by the higher frequency of more aggressive undifferentiated forms in this area. Subsequently, the Health & Healthcare Services Board of the Directorate-General of Public Health for the Principality of Asturias recorded 517 cases of TC in the period 1982–1993, distributed mainly throughout the southern and eastern municipal areas of the region [51].

There are hardly any studies that address TC status or prevalence of goiter in the Canary Islands in the years preceding this study. In one undertaken in 1986 in Las Palmas Province, it was the Island of Gran Canaria that registered the highest incidence of goiter, principally in mountainous areas. Puerto del Rosario on Fuerteventura (the town with the highest mortality rate in our study) and Santa Lucía on Gran Canaria were two of the districts having a greater percentage of admissions due to goiter. Nearly 10% of such cases developed into carcinomas, mainly follicular [52]. Furthermore, in the period 1993–1995, this tumor's incidence rate in the Canary Islands was far higher among women than among men (7.05 and 1.65 per 100,000 person-years respectively)[18].

There are other Spanish regions which have traditionally been regarded as endemic goiter areas, yet nonetheless failed to register such a marked risk in TC mortality in our study. Pre-eminent among these are certain mountain districts in the Pyrenees, Cadiz, Huelva, Seville and Las Hurdes [29,53]. This finding has led us to consider the possible existence of causes, unknown until now, which might account for the geographic pattern observed in this study.

The best-evidenced etiologic factor implicated in TC is ionizing radiation. Medical irradiation of the head and neck, particularly during childhood, has been linked to increases in this type of tumor [32,54-56]. Indeed, it has been estimated that the risk of a thyroid nodule proving malignant is 30%–40% if the person in question underwent radiation during his/her childhood [32]. Formerly, it was common practice to irradiate in order to treat benign childhood affections (such as ringworm of the scalp, recurrent tonsillitis, acne, or enlargement of the thymus) or even cancer, fundamentally of head and neck. In this respect, it is noteworthy that the geographic patterns for TC bear some similarity with the patterns displayed by tumors of the buccal cavity and pharynx, with the latter being more common in west Andalusia, the Canary Islands and the Basque Country [57]. To our knowledge, however, there is no evidence of the existence of differences in clinical practice in Spain. Moreover, the geographical distribution of natural radiation in Spain does not correspond to the patterns plotted for highest mortality in our study [58].

Furthermore, the possible influence of genetic factors on the distribution of TC mortality should not be ruled out. These factors are particularly important in medullary thyroid cancer, where one third of cases are linked to hereditary syndromes [17]. However, nonmedullary tumors can also be associated with the so-called syndrome of familial nonmedullary thyroid cancer [59]. In this regard, the areas concerned -relatively isolated and virtually inaccessible until a few years ago- might possibly be characterized by a certain tendency towards endogamy.

## Conclusion

In conclusion, municipal maps can be a useful tool from a public health standpoint, since they allow for detection of risk areas that might otherwise be masked at a provincial level and identification of possible etiologic factors. The geographic study of TC mortality at a municipal level has enabled us to detect a very marked pattern in the north-west of the country and in the Canary Islands, areas traditionally designated as goiter-endemic. The hypothetical presence of more aggressive histologic types in such areas, as well as the possible action of other unknown genetic or environmental factors might be possible etiologic hypotheses to be borne in mind in future geographic studies.

## Competing interests

The author(s) declare that they have no competing interests.

## Authors' contributions

VL conceived the idea and wrote the manuscript; RR and DGB carried out the statistical analysis; BPG and NA made contributions to statistical analyses and interpretation of results, and revised the manuscript for important intellectual content; GLA and MP designed the study, contributed to manuscript writing, and revised it for important intellectual content. All authors contributed to the final version of the manuscript.

## Pre-publication history

The pre-publication history for this paper can be accessed here:



## Supplementary Material

Additional data file 1 Spanish Autonomous Regions and provinces. a map showing the respective Spanish Autonomous Regions and provinces is provided. Click here for file
