# Supplementary figures and images for: Municipal mortality due to thyroid cancer in Spain
Source: BMC Public Health. 2006 Dec 15;6:302. doi: 10.1186/1471-2458-6-302 (PMC1764886; doi:10.1186/1471-2458-6-302)

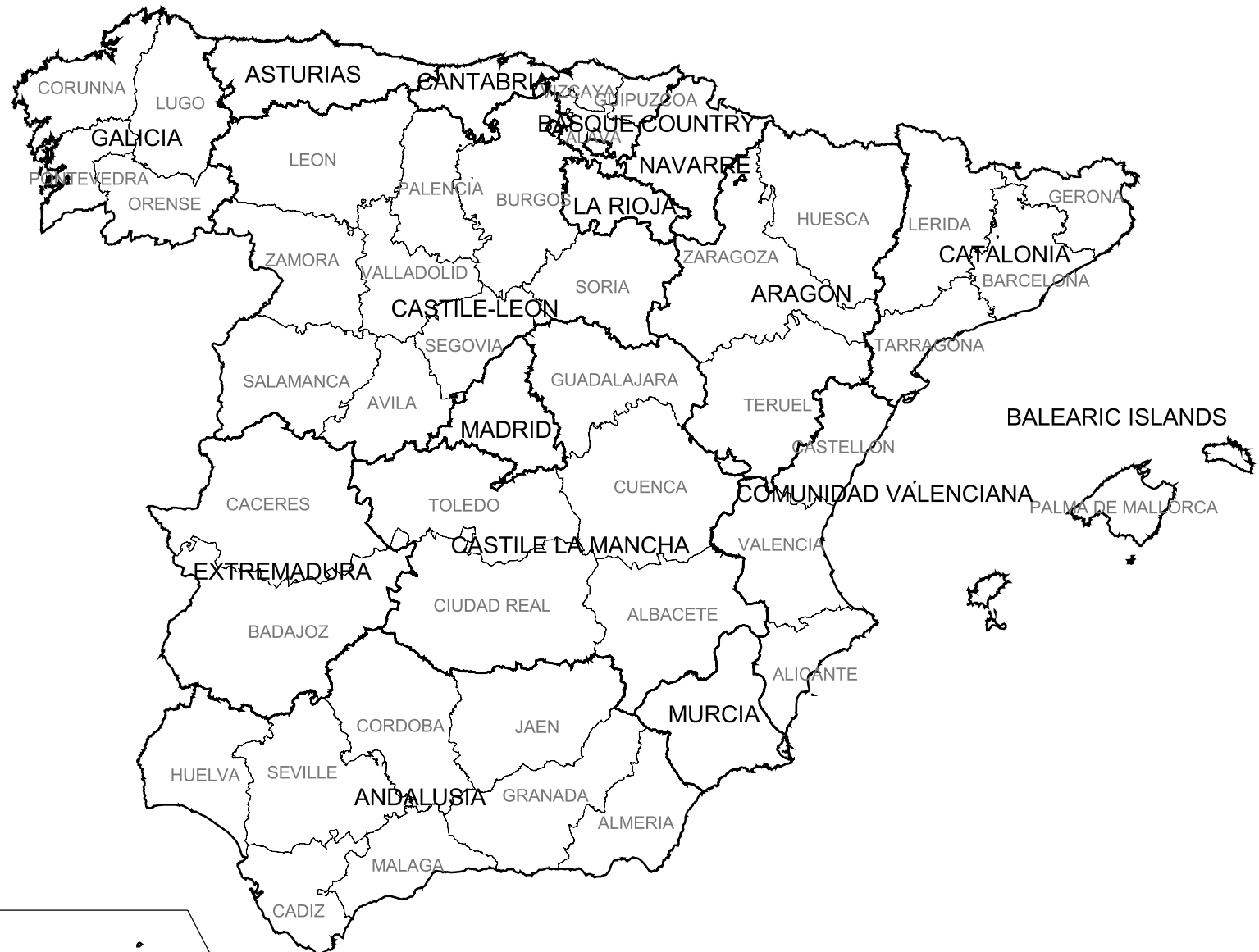

CEUTA

CEUTA Y MELILLA

MELILLA

CANARY ISLANDS

SANTA CRUZ DE TENERIFE

LAS PALMAS DE GRAN CANARIA

Supplement: Additional data file 1 — Spanish Autonomous Regions and provinces. a map showing the respective Spanish Autonomous Regions and provinces is provided. [file 1471-2458-6-302-S1.pdf]
